# Interacting Factors Driving a Major Loss of Large Trees with Cavities in a Forest Ecosystem

**DOI:** 10.1371/journal.pone.0041864

**Published:** 2012-10-05

**Authors:** David B. Lindenmayer, Wade Blanchard, Lachlan McBurney, David Blair, Sam Banks, Gene E. Likens, Jerry F. Franklin, William F. Laurance, John A. R. Stein, Philip Gibbons

**Affiliations:** 1 Fenner School of Environment and Society, The Australian National University, Canberra, Australian Capital Territory, Australia; 2 Australian Research Council Centre of Excellence for Environmental Decisions, and The National Environment Research Program, The Australian National University, Canberra, Australian Capital Territory, Australia; 3 Cary Institute of Ecosystem Studies, Millbrook, New York, United States of America; 4 School of Environmental and Forest Science, University of Washington, Seattle, Washington, United States of America; 5 Centre for Tropical Environmental and Sustainability Science and School of Marine and Tropical Biology, James Cook University, Cairns, Queensland, Australia; Australian Wildlife Conservancy, Australia

## Abstract

Large trees with cavities provide critical ecological functions in forests worldwide, including vital nesting and denning resources for many species. However, many ecosystems are experiencing increasingly rapid loss of large trees or a failure to recruit new large trees or both. We quantify this problem in a globally iconic ecosystem in southeastern Australia – forests dominated by the world's tallest angiosperms, Mountain Ash (*Eucalyptus regnans*). Tree, stand and landscape-level factors influencing the death and collapse of large living cavity trees and the decay and collapse of dead trees with cavities are documented using a suite of long-term datasets gathered between 1983 and 2011. The historical rate of tree mortality on unburned sites between 1997 and 2011 was >14% with a mortality spike in the driest period (2006–2009). Following a major wildfire in 2009, 79% of large living trees with cavities died and 57–100% of large dead trees were destroyed on burned sites. Repeated measurements between 1997 and 2011 revealed no recruitment of any new large trees with cavities on any of our unburned or burned sites. Transition probability matrices of large trees with cavities through increasingly decayed condition states projects a severe shortage of large trees with cavities by 2039 that will continue until at least 2067. This large cavity tree crisis in Mountain Ash forests is a product of: (1) the prolonged time required (>120 years) for initiation of cavities; and (2) repeated past wildfires and widespread logging operations. These latter factors have resulted in all landscapes being dominated by stands ≤72 years and just 1.16% of forest being unburned and unlogged. We discuss how the features that make Mountain Ash forests vulnerable to a decline in large tree abundance are shared with many forest types worldwide.

## Introduction

Large trees with cavities play critical roles in forest, agricultural and urban ecosystems worldwide [1]–[6]. These roles include: storing carbon [7]–[10]; creating distinct microenvironments characterized by high levels of soil nutrients, plant species richness and structural complexity [7], [11]; and providing nesting and sheltering habitat for numerous animal species (>350 mammal species globally) [12], [13] including up to 30% of the vertebrate biota in a given vegetation type [3], [14], [15]. Large trees with cavities can take a prolonged time to develop – more than century in Douglas-fir (*Pseudostuga menziesii*) trees in western North America [3] and the vast majority of Australian eucalypt species [14] and 200 years in European Pedunculate Oak (*Quercus robus*). However, many ecosystems worldwide are increasingly characterized by the rapid loss of large trees with cavities, a failure to recruit new trees with cavities, or both [1], [11], [16]–[20]. Many kinds of human disturbances cause this problem, including recurrent logging, altered fire regimes, grazing by domestic livestock, and the impacts of exotic plants. The loss is global, occurring in North America [21]–[23], South America [24], [25], Europe [26], [27], Asia [28], and Australia [29], [30].

It is vital to better understand the processes driving the population dynamics of large cavity-bearing trees and the factors influencing those dynamics given their important roles, the extended period required for their development, and increasing concern about their rarity in many ecosystems [13], [31].

Using long-term datasets comprising repeated measurements of large trees with cavities, we quantify, for the first time, the combined and inter-acting influences of natural and human disturbances, site productivity, climate and other factors on large tree population dynamics focusing on the Mountain Ash (*Eucalyptus regnans*) forests of the Central Highlands of Victoria. This iconic ecosystem incorporates the world's tallest flowering plants [32], includes stands with the highest reported above-ground biomass globally [8], and provides habitat for high-profile globally endangered cavity-dependent fauna such as Leadbeater's Possum (*Gymnobelideus leadbeateri*), which is virtually confined to the ash forests of Victoria [33].

We pose three questions related to quantifying the rates of, and factors influencing, the dynamics of large living and dead trees bearing cavities:

- *What are the relationships between fire severity and the mortality and collapse of large cavity trees?* Approximately 50% of our study region was burned in major wildfires in 2009 [34]. Because Mountain Ash trees are considered fire sensitive [35], we predicted that most living cavity trees would be killed on burned sites. However, we anticipated that death rates would be lower on sites subject to moderate rather than high severity fire. In addition, we hypothesized that individual larger diameter and taller trees would be more likely to survive because they have thicker bark and the canopy has a higher probability of being above flame height [36]. Wildfire also can be an important process generating new cavity trees [37], including in Australian eucalypt forests (e.g. [38]) and therefore we postulated that new large cavity trees would be recruited on our field sites subject to wildfire in 2009.
- *How are tree mortality and collapse related to site productivity (reviewed by* [*31*] *) and climatic conditions* *[39]*–*[41]* *?* A prolonged hot drought occurred prior to the major wildfires in our study area in 2009 [34], [42]. Mountain Ash occupies sites with >1200 mm of rainfall annually [43] and has a limited capacity to regulate transpiration, making it potentially sensitive to moisture stress [44], [45]. For this reason, we postulated that rates of tree mortality in the five years preceding the 2009 fire would be significantly higher than what they were at the beginning of the data collection period (1998–2003).
- *What is the influence of site-level stand age and topographic factors (slope, elevation and aspect) on large cavity trees?* Microclimatic conditions, such as wind speed and temperature, can vary markedly across forest landscapes [46], [47] and between old and young stands (e.g. [48]). We also explored relationships among tree-level attributes such as tree condition, height and diameter and the probability of mortality and collapse of large cavity trees. Earlier work on large cavity trees has underscored the importance of the factors on tree decline and collapse in a range of forest systems (e.g. [49]–[51]), including Mountain Ash forests [52].

Projections of temporal changes in the abundance of large trees with cavities for the next 50 years were possible based upon these analyses and quantification of some of the key drivers of large tree population dynamics. They also were the developmental basis for a new conceptual model of the relationships among tree-, stand- and landscape-level drivers that have both accelerated the loss of existing trees with cavities and created barriers to the recruitment of new ones.

We intend for this paper to contribute to the scientific understanding of the dynamics of populations of large cavity-bearing trees and the conservation and management of an array of forest types worldwide, including several that are similarly vulnerable to Mountain Ash forests in declines in abundance of large cavity-bearing trees.

### Large trees with cavities in Mountain Ash forests

Large living and dead trees with cavities are a critical nesting and denning resource for >40 species of native vertebrates in Mountain Ash forests [53], including the endangered Leadbeater's Possum. Primary cavity-excavating species such as woodpeckers are absent in Australia and development of large trees with cavities requires long time periods because it occurs through the activities of termites and fungi [14]. Cavities begin appearing in Mountain Ash trees that exceed 120 years old [54] but the large hollows that provide nest sites for most birds and mammals generally do not occur until trees exceed 190 years [53].

Mountain Ash trees may live for up to ∼500 years – which is 380 years beyond the time when cavities regularly appear [55]. After death, large dead trees with cavities usually remain standing for 10–75 years [56] and continue as important denning and nesting sites for many cavity-dependent animals [14].

The greatest abundance of living cavity-bearing trees is typically found in old growth forests (i.e. stands exceeding 200 years old) [57]. However, living and dead cavity trees also occur in much younger stands of Mountain Ash. These cavity trees are biological legacies (*sensu* [58]) of a previous old-growth stand, which survive a natural (typically fire) or human (i.e. trees retained during logging operations) disturbance, thereby structurally enriching a young regenerating cohort [59].

Disturbances play a pivotal role in influencing the recruitment, decay and collapse of large trees with cavities in Mountain Ash forest. Fire is the principal form of natural disturbance [35]. Because Mountain Ash is fire-sensitive and wildfires almost always include severely burned areas with high tree mortality, these places support regeneration of new cohorts of Mountain Ash trees [35]. If stand-replacing wildfires recur frequently (<20–30 years), seeds of Mountain Ash are not available because young trees do not have time to mature [44] and other species, such as wattle (*Acacia* spp.), will replace Mountain Ash [33].

Clearcut logging is the main human disturbance in Mountain Ash forest influencing the population dynamics of large cavity-bearing trees. All merchantable trees within an area of 15–100 ha are clearcut in a single operation. The logged area is then subjected to a high-intensity slash-burn to create a bed of ashes in which the regeneration of a new stand of eucalypts occurs, often by artificial reseeding. Current logging prescriptions allow for the retention of 10 trees per 15 ha of harvested forest. However, extensive surveys indicate that these trees often are either destroyed in the regeneration burn or collapse soon after [33], [60].

The vast majority of Mountain Ash landscapes in the Central Highlands are now dominated by young stands (<73 years old) because of intensive logging of large areas and large intense wildfires in 1939, 1983 and 2009. Older stands (originating before 1900) are rare but are fully protected from logging [61]. Only ∼1886 ha of old growth forest – just 1.16% of the 161,200 ha Mountain Ash landscape – remains following the last 100 years of logging and wildfire (Victorian Department of Sustainability and Environment unpublished data 2012).

### Definition of a large cavity tree

We define a large cavity-bearing tree as any tree >0.5 m in diameter at breast height (dbh) containing one or more obvious hollows (based on repeated ground-based observations using binoculars). We assigned each cavity in a large cavity tree to one of three categories: (1) a fissure is any narrow crack in the tree trunk >1.5 cm in diameter and >3 cm long; (2) a hole is any opening in the tree trunk >4 cm wide; and (3) a hollow branch has an opening >4 cm in diameter. We recorded the number of observed fissures, holes and hollow branches in each tree. All of the large cavity trees in this study were eucalypts – primarily Mountain Ash but also some Alpine Ash (*Eucalyptus delegatensis*), Shining Gum (*E. nitens*) and Mountain Grey Gum (*E. cypellocarpa*). No large cavity trees were understory plants such as Silver Wattle (*Acacia dealbata*), Mountain Hickory Wattle (*A. obliquinervia*), Forest Wattle (*A. frigescens*), Blackwood (*A. melanoxylon*), Myrtle Beech (*Nothofagus cunninghamii*) and Southern Sassafras (*Atherosperma moschatum*).

Importantly, the large trees with cavities we have carefully monitored in this study have been mapped and georeferenced with a GPS and then marked using permanent painted numbers and metal tags. This has enabled us to readily revisit and remeasure the same large cavity trees in our marked population and followed the fates of each one.

Our ground-based surveys using binoculars may have overlooked some cavities and recorded others that were in fact unsuitable for use by animals (see [25], [62]). However, we adhered strictly to our initial definition of a large cavity tree and employed the same field methods for measuring cavities since commencing work in 1983 [33], [63]. This was essential to maintain the statistical and ecological integrity of the long-term data record (see [64]).

## Methods

### Study area

**Figure 1:**
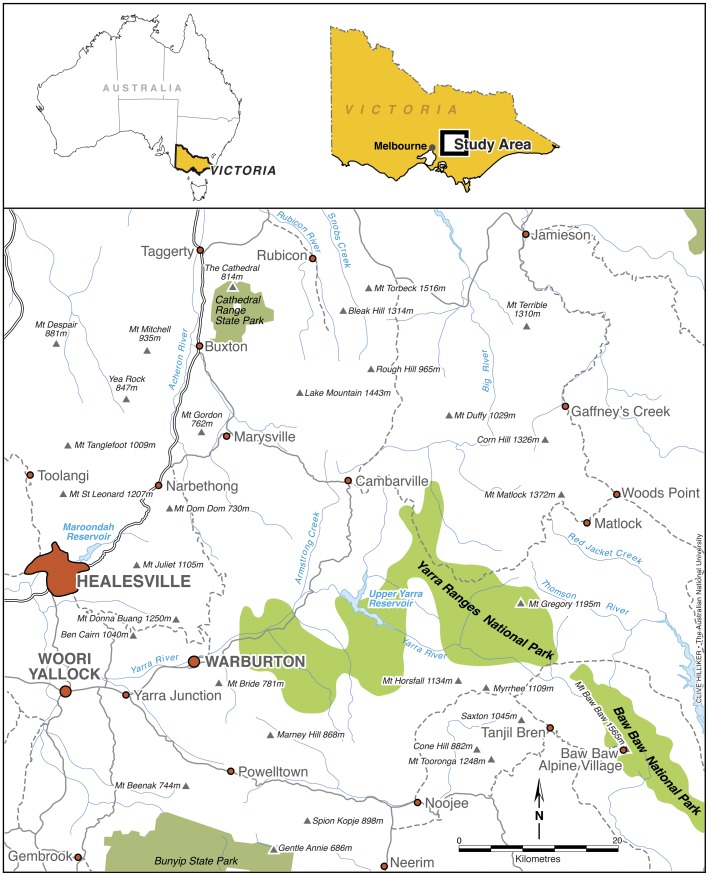
The study area in the Central Highlands of Victoria, south-eastern Australia.

The study area lies ∼120 km north-east of Melbourne in south-eastern Australia and covers approximately 60 km×80 km (37°20′–37°55′S and 145°30′–146°20′E; Figure 1). Mountain Ash forests are characterized by mild, humid winters with occasional periods of snow. Summers are generally cool. Mountain Ash typically occurs at altitudes between 400 and 900 meters in our study area [65]. Further information on the study area is available in [33].

Understory tree and shrub layers in Mountain Ash forests can be well developed and support a range of plant species [66], [67]. Prominent species include Myrtle Beech, Southern Sassafras and four species of wattle [33].

No specific permits were required for the described field studies. The relevant permissions to enter the government land where the studies were undertaken were given by Parks Victoria, Melbourne Water, and the Victorian Department of Sustainability and Environment. All native animal species and native woodland vegetation are protected in Australia, including endangered birds and plants. Our studies were observational investigations and no plants or animals were harmed in any way.

### Datasets

**Figure 2:**
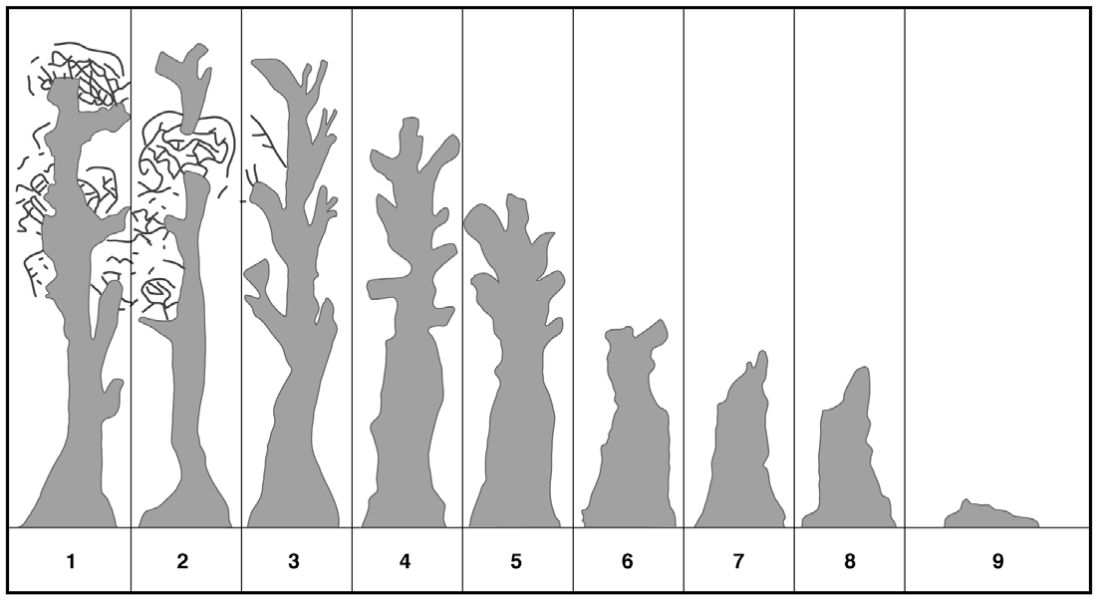
Sequential development of tree forms in Mountain Ash trees. Form 1: Mature, living tree; Form 2: Mature living trees with a dead or broken top; Form 3: Dead tree with most branches still intact; Form 4: Dead tree with 0–25% of the top broken off; branches remaining as stubs only; Form 5: Dead tree with top 25–50% broken away; Form 6: Dead tree with top 50–75% broken away; Form 7: Solid dead tree with ≥75% of the top broken away; Form 8: Hollow stump. In subsequent surveys we added a ninth category – Form 9: Collapsed tree.

We used a suite of datasets in our investigation. First, we measured the condition (*sensu* Figure 2) of 1129 large trees with cavities on 156 permanent 1- ha field sites on a repeated basis in 1997, 2006, 2009, 2010 and 2011. This dataset was the primary one used in this study and we describe it in detail in the following section. Second, we calculated measures of productivity for each of the 156 sites. Third, we assembled long-term temperature and rainfall data (http://www.bom.gov.au/index.shtml?hdrc) to determine if long-term rates of tree death and collapse were associated with temporal patterns in regional climate conditions. Fourth, we calculated standardized death and mortality rates between 1997 and 2011 for our 156 sites and compared them against historical rates of tree death and collapse for other datasets we gathered in Mountain Ash forests, *viz*: **(i)** 286 large cavity trees on 29×3 ha sites measured in 1983, 1988, 1993 and 2007 [56], **(ii)** 744 large cavity trees measured on 109 sites each of 3 ha in 1988 and 1993 [52], and **(iii)** 399 large cavity trees measured in 1998 and 1993 on 49 linear strips of forest retained adjacent to logging cutblocks [52].

### Site-level measurements and derived climate and productivity measures

We established 156 1 hectare permanent monitoring sites in 1997. The sites supported 1 to 31 large trees with cavities (mean 7.3, median 6.0). The sites were dominated primarily by Mountain Ash but also included some Alpine Ash and Shining Gum trees.

Our sites encompassed a variety of stand ages including those dating from the mid-1700s, mid-1850s, the early 1900s (1905, 1919, 1926 and 1932), 1939 and ∼1983. We ensured there was a minimum of eight sites in each of these forest age cohorts but we examined more stands in 1939-aged forest than other age cohorts. This difference occurred because at the time we commenced this study, forests regenerating after the extensive 1939 conflagration dominated the Central Highlands of Victoria and comprised more than 70% of the ash-type eucalypt forest in the region [33]. We measured the slope of each site with a clinometer and extracted data on site aspect from a 20 m scale Digital Elevation Model (DEM). We assigned aspect to one of the following categories: north, east, south, and west.

We derived values for climate and productivity for each of our 156 field sites. One of these was the Topographic Wetness Index (TWI) [68], which gives a measure of relative position in the landscape, and thus potential water distribution. Calculation of TWI requires a DEM that has hydrological integrity, and we used the *ANUDEM 5.2* algorithm (http://fennerschool.anu.edu.au/research/publications/software-datasets/anudem) to generate a DEM of our study region at a grid resolution of 20 m. For each cell, the size of the catchment that flows to it was divided by its width, adjusted geometrically by the aspect of inflow direction. This ‘specific catchment’ was then divided by the cell's local slope. Lower values indicate ridges and upper slopes that have no, or small, contributing catchment, with values then increasing through lower slopes, valley flats, and drainage lines.

The GROWEST model (http://fennerschool.anu.edu.au/research/publications/software-datasets/growest) [69] assesses site productivity by integrating the effects of moisture, temperature and solar radiation, and has been adapted as GROCLIM (http://fennerschool.anu.edu.au/research/publications/software-datasets/anuclim) to use monthly mean climate data. The Moisture Index (MI) component is the most likely to vary across small geographic areas, so we calculated long-term mean MI (1976–2005) values at each grid cell in our study region. The DEM was used to estimate monthly mean precipitation and evaporation to calculate a running water balance over the year, assuming a default available soil water holding capacity of 150 mm. Values were scaled from 0 (dry), typically occurring in late summer, to 1 (saturated), above which runoff occurs, typically in late winter.

### The 2009 wildfires

In February 2009, a major conflagration termed the ‘Black Saturday’ wildfires burned our study region. We subsequently completed on-ground surveys of each site to quantify fire severity on a scale of 1 (no fire) to 5 (very high fire severity in which the crowns of the overstorey trees had been totally consumed by the fire). Eighty-eight of our 156 permanent sites were not affected by fire, 46 experienced a moderate fire, and the remaining 22 experienced a severe fire. This corresponded to 623, 276 and 230 large trees with cavities consumed, respectively. The fire occurred before the 2009 tree assessment; thus our 1997 and 2006 data were pre-fire and the 2009, 2010 and 2011 assessments were conducted post-fire.

### Tree-level measurements

In 1997 we mapped and permanently marked all 1129 large trees with cavities on the 156 field sites. We assigned each tree to one of eight tree form or decay classes based on readily observable external characteristics (see Figure 2). Thus, our population of marked trees encompassed both living and dead stems (Figure 2). We completed a range of other measurements of all marked trees including tree diameter (measured with a diameter tape at 1.3 m above the ground) and tree height (measured with a range-finder).

Each time we re-surveyed a given field site, we completed an additional 3 hour reconnaissance in which all overstorey eucalypt trees on each site were inspected with binoculars. We completed these surveys as part of a detailed vegetation surveys on all 156 field sites and we used these surveys to determine if any new cavity trees had been recruited since the previous survey.

### Statistical analyses

We conducted statistical analyses of tree death and tree collapse in four stages. First, we analyzed death rates of large living trees with cavities. That is, we quantified the rates of death among trees that were alive at the start of the study (Forms 1 and 2, see Figure 2) and then constructed statistical models of the tree and site-level factors significantly influencing mortality. Second, we quantified the rates of collapse of both living and dead large trees with cavities and developed statistical models of the tree and site-level factors significantly influencing the probability of tree collapse. Third, we constructed transition probability matrices of the movement of large trees with cavities through different stages of tree decay. Fourth, we made projections of the future abundance of large trees with cavities.

#### Mortality and collapse

We investigated the factors influencing tree death and collapse using methods similar to those described in [56]. We briefly summarize the key points of the analysis but refer readers to [56], [70] for further details. For simplicity, we describe the analysis approach for tree death, but we also applied the same methods to our data on tree collapse.

Let 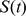 be the survival function of a tree with hollows, that is, the probability that a tree is alive after time, t. The key feature of the model is the link between the survival function of the i^th^ tree and its linear predictor, 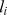, is given by:
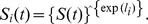



The linear predictor, 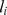, comprises the potential predictors measured at both the tree and site levels. The probability of death, denoted by 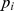, in an interval of length 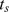 is given by:
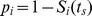
and thus:
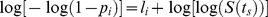
which can be viewed as a generalized linear model with a binomial distribution and a complementary log-log link function (see [70]).

We embedded the interval-censored survival model [70] into a Generalized Linear Mixed Model (GLMM) framework [71]. That is, we combined the binomial distribution for the response variable with complementary log-log link function with random effects for site and tree within site. Following the approach applied by [56], we controlled for the length of time between measurement periods by adjusting the integrated hazard rate by the time interval; more details are provided in Supplementary Information S1.

To explore relationships between tree mortality and climate variables such as the derived moisture index, we used logistic regression modelling in which we controlled for the length of time between repeated measurements of trees. Given that Mountain Ash is a mesophyte that occupies sites with >1200 mm of rainfall annually [43], has limited ability to regulate transpiration and is therefore potentially most sensitive to moisture stress [44], [45], we focused this part of our analysis on a derived moisture index for periods of lowest available moisture, January-March.

The basic design of our study encompassed a two-way layout with fire severity (none, moderate, severe) and time period (1997–2006, 2006–2009, 2009–2010, 2010–2011). However, given the timing of the 2009 fire, fire severity could not be included in the first time interval. Thus, rather than having a complete 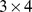 design with 12 cells, our design comprised 10 cells (see Table 1). Our basic design had a period effect plus design variables for fire severity in the subsequent time periods. We modeled our 10-cell design with 3 degrees of freedom for period and 6 degrees of freedom for severity×time period (2 fire severities
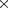
3 time periods) to capture the fire severity by time period interaction. Note that our design did not include a main effect for fire severity.

**Table 1:** The overarching modelling framework used to quantify relationships between period and fire and the mortality and collapse of large cavity trees.

	Time period			
Fire severity	1997–2006	2006–2009	2009–2010	2010–2011
**No fire**	C	C+P2	C+P3	C+P4
**Moderate fire**		C+P2+MF.P2	C+P3+MF.P3	C+P4+MF.P4
**Severe fire**		C+P2+SF.P2	C+P3+SF.P3	C+P4+SF.P4

C is the overall constant in the model; P2, P3 and P4 are the period effects; MF.P2 is the effect of moderate fire in period 2; SF.P2 is the effect of severe fire in period 3, etc.

We entered all variables into the model and subsequently eliminated terms via a backward elimination procedure using the 5% level. However, the design variables given in Table 1 were not subjected to the backward elimination procedure. Due to small numbers of observations in some groups, not all two-way interactions could be estimated. However, we retained those interactions in the model that met the 5% level. We tested categorical variables found to be significant (P<0.05) with Fisher's protected Least Significant Differences to determine which levels differed as recommended by Milliken and Johnson [72].

#### Transitions through different decay stages

We quantified the rates of transition among tree forms between 1997–2011 by computing the fraction of trees of a given form in 1997 that either remained in the same class or progressed to more decayed form. We conducted this operation for each of three fire severity classes (no fire, moderate fire, severe fire). We estimated the transition probability matrices using the following combined classes of tree forms: 1–2, 3–5, 6, 7, 8, 9 (see Figure 2). In addition, we compared the transition probability matrices using log-linear modelling, keeping the appropriate margins of the table fixed [73].

#### Historical rates of tree death and collapse on unburned sites

We calculated standardized death and collapse rates for sites measured between 1997 and 2011 and compared them against “historical rates of tree mortality and tree collapse for all of our large cavity tree datasets using chi-squared goodness of fit tests and controlling for the duration of the observation period. We excluded the trees measured between 1997 and 2011 on 68 of our 156 sites that were burned in 2009 because of the overwhelming effects of fire on tree mortality and collapse on these sites. To assess the possible differences in the decay process over time, we compared two 14-year transition matrices: 1993–2007 and 1997–2011.

#### Projections of future availability of large trees with cavities

We used the transition probability matrices for temporal changes in tree forms to make projections of the future abundance of large trees with cavities until 2039 and to 2067. We choose these times because: **(1)** they corresponded to a multiple of the length of time of the 14-year measurement interval (between 1997 and 2011), and **(2)** 2067 is the approximate time at which existing 73-year old trees in stands dominated by unburned 1939 regrowth trees reach 120 years old and regularly begin to develop cavities [53]. We employed a parametric bootstrapping procedure to estimate the prediction standard errors using 10,000 samples. For these projections, we also assumed no further wildfires between 2011 and 2067, and no logging on any of our 156 long-term sites where we quantified tree death and collapse. In addition, we assumed our 156 sites were representative of the broader Mountain Ash forest estate *per se*. However, we were acutely aware that, for example, our number of old growth sites (18 of 156 sites = 11.5%) was substantially greater than the actual proportion of old growth forest that currently characterizes Mountain Ash forests (1.16%; see below). Therefore, our projection of the future abundance of large cavity trees was likely to be optimistic.

## Results

**Figure 3:**
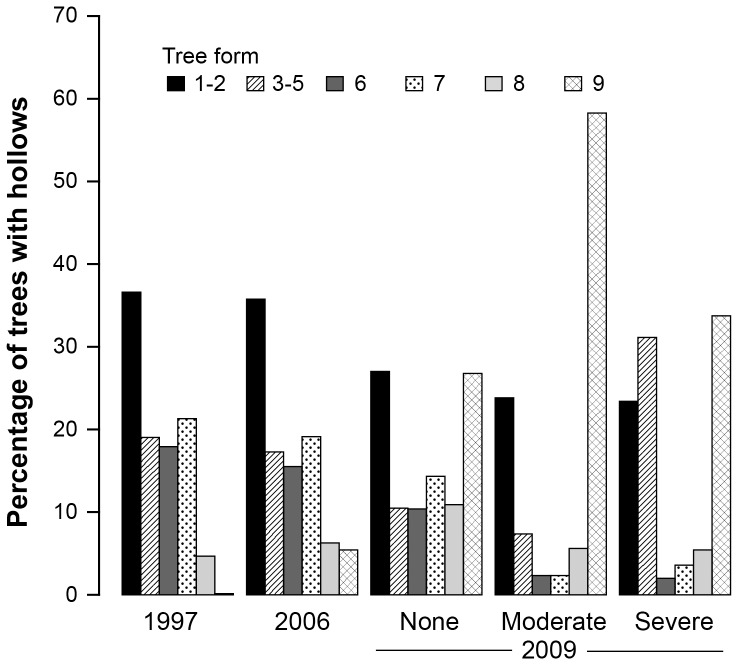
Temporal changes in the numbers of trees of different forms. The relative composition of populations of large trees with cavities in different forms (*sensu* Figure 2) is shown for 1997–2009.

We found that the number of large trees with cavities in different forms (sensu Figure 2) in 1997, 2006 and 2009 (the first year post fire) was characterized by a drastic post-fire shift in the composition of decay classes (Figure 3). We also identified a substantial shift in the number of large trees with cavities that collapsed in 2009 on our unburned sites (Figure 3).

We found no recruitment of new large trees with cavities on any of our 156 field sites measured repeatedly between 1997 and 2011.

### Tree death

Rates of death of large trees with cavities were highest in the 2006–2009 period and particularly on sites subject to high severity fire (Table 2). Severe fire had a highly significant (P<0.001) effect on tree death (Supplementary Information S2). A total of 79.4% of large living trees with cavities died on sites subject to high-severity fires whereas the equivalent value for moderate fire-severity sites was 36.8% (Table S1). We also found that the probability of cavity tree death was significantly influenced by: **(1)** tree species (P<0.001), with Shining Gum exhibiting lower death rates than the other species, and **(2)** a tree height×severe fire 2006–2009 interaction (P<0.001), with taller trees less likely to die, for all time periods and fire classes with the exception of the severe fire in the 2006–2009 period (see Supplementary Information S2). After the 2009 fire, the rates of tree death on sites subject to moderate fire were comparable to those on unburned sites but rates on severely burned sites remained elevated in both the 2010 and 2011 measurement periods compared with unburned sites (Table 2; Supplementary Information S2).

**Table 2:** Percentage rates of mortality of large cavity trees by fire category adjusted (standardized) for the duration of each measurement period.

	Time Period			
Fire Severity	1997–2006	2006–2009	2009–2010	2010–2011
No Fire	0.24 (9/414)	4.00 (21/182)	1.24 (2/161)	0.63 (1/159)
Moderate		13.51 (30/85)	5.45 (3/55)	3.85 (2/52)
Severe		22.60 (74/138)	34.38 (22/64)	30.95 (13/42)

Values in parentheses are the numbers of large cavity trees that died over total measured trees for each time period. The first measurement in the 2009–2010 period was in April 2009, two months after the 2009 wildfire. The standardized mortality rate, 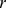, was calculated by 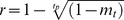 where 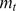 is fraction of trees experiencing mortality during a time period of length 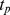.

### Tree collapse

We found that rates of cavity tree collapse were highest between 2006–2009 for all fire severity classes, but particularly on moderately burned sites (Table 3). The elevated rates of collapse experienced during the 2006–2009 period returned to rates similar to those observed in the previous period (i.e. 1997–2006). In addition to time period and fire severity, three covariates significantly influenced the probability of cavity tree collapse (Supplementary Information S3): **(1)** trees of form 1–2 (see Figure 2) experienced significantly (P<0.001) lower rates of collapse compared to all other forms and trees of form 8 experienced significantly (P<0.001) higher rates of collapse than trees of forms 3–7, **(2)** large trees with cavities within old growth stands were significantly (P = 0.009) less likely to collapse than trees within 1939-aged stands and ∼20-year old stands, and **(3)** trees on high productivity sites were marginally significant (P = 0.074) more likely to collapse than trees on low productivity sites (Supplementary Information S3).

**Table 3:** Percentage rates of collapse of large cavity trees by fire category adjusted (standardized) for the duration of each period.

	Time Period			
Fire Severity	1997–2006	2006–2009	2009–2010	2010–2011
No Fire	0.64 (63/1129)	6.78 (110/579)	3.62 (17/469)	3.76 (17/452)
Moderate		25.54 (155/264)	0.92 (1/109)	1.85 (2/108)
Severe		12.97 (76/223)	2.72 (4/147)	0.00 (0/143)

Values in parentheses are the numbers of collapsed large cavity trees over the total measured trees for each time period. The first measurement in the 2009–2010 period was in April 2009, two months after the 2009 wildfire. The standardized collapse rate, 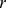, was calculated via 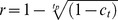 where 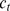 is the fraction of trees experiencing collapse during a time period of length 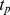.

### Transitions between trees in different condition categories for different fire classes

We show in Appendix B the transition probability matrices for each of the three fire severity classes (unburned, moderate, and severe). Log-linear modelling revealed a highly significant difference 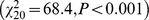 among the transition probability matrices indicating that the transition process was markedly different between unburned sites and sites experiencing moderate fire and severe fire (Table S1).

There were several important features of the transition matrices that are consistent with the results for tree death and collapse that we outlined above: **(1)** High levels of mortality of living trees with cavities on unburned sites (14% between 1997 and 2011). **(2)** Very high levels of mortality on sites burned at high severity (79%). **(3)** Very high levels of loss of dead trees with cavities on burned sites (irrespective of fire severity), ranging from ∼60% of trees in forms 3–5 to 100% of trees in form 8 (Table S1).

### Historical rates of tree death and tree collapse on unburned sites

**Figure 4:**
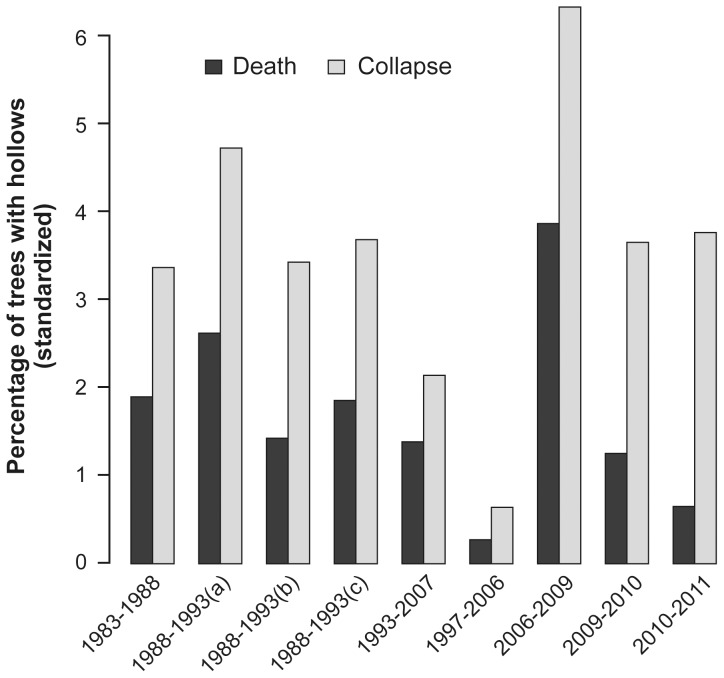
Historical death and collapse rates of trees on sites which did not experience wildfire in 2009. Note (a) corresponds to a dataset comprised of 286 large trees with cavities on 29 sites measured in 1988 and1993, (b) refers to 744 large trees with cavities measured on 109 sites in 1988 and 1993, and (c) corresponds to a dataset comprised of 399 large trees with cavities on 49 linear strips measured in 1993 and 1998.

We found highly significant between-measurement period differences on unburned sites for standardized tree death rates 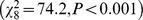 and standardized tree collapse rates 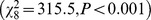 (Figure 4). Standardized death rates were: **(1)** significantly lower in the 1997–2006 period compared to the historical rates, and **(2)** significantly higher in 2006–2009 than for all other periods except 1988–1993(a) (see Figure 4).

For standardized collapse rates, we found: **(1)** the 1997–2006 period was characterized by a significantly lower collapse rate than historical periods, and, **(2)** the 2006–2009 period had a significantly higher collapse rates than other periods except 1988–1993(a) (see Figure 4).

We compared the 14-year probability transition matrix computed from 1993–2007 to the one computed for 1997–2011 but found no evidence of a significant difference between them 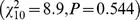.

We completed extensive analyses of relationships between climate variables calculated for the corresponding measurement period and standardized death and collapse rates. We found no significant relationships, although there was a marginal association between the standardized death rate and the value for the minimum moisture index for January to March (P = 0.074). That is, higher death rates were evident when values for the moisture index were low (data not shown).

### Projections of the future abundance of large trees

**Figure 5:**
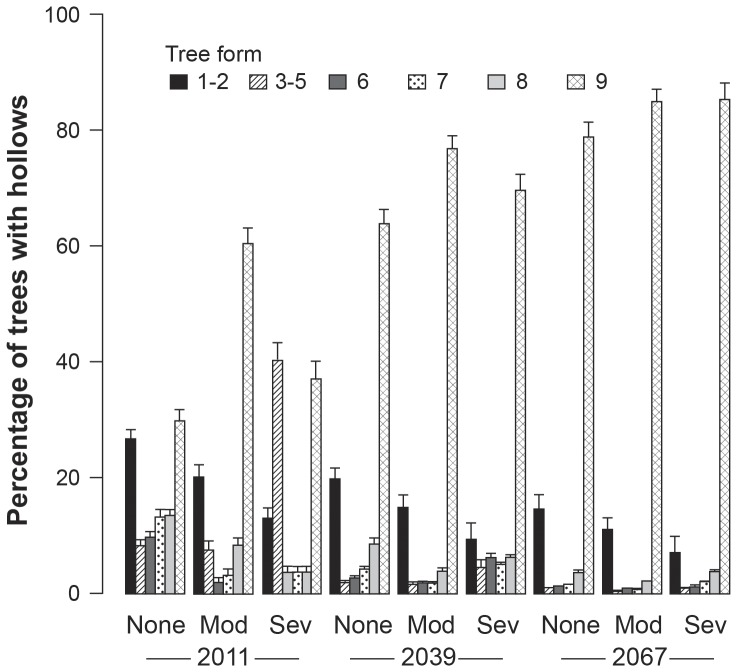
Projected relative composition of forms of large trees with cavities in 2011, 2039 and 2067. The latter date is when existing 72 year old trees will reach 120 years of age and regularly begin developing cavities – see text. This assumes no logging and no further fire on the 156 sites used to make the projection. In addition, we assume there are no changes in moisture index.

Based on the 1997–2011 transition probability matrix, we projected that by 2039 most sites and particularly those severely burned in 2009 will be overwhelmingly characterized by collapsed trees with cavities (Figure 5). Additionally we project a paucity of standing large trees with cavities on unburned sites and on sites subject to moderate severity fire (Figure 5). These patterns were further magnified by 2067.

## Discussion

Large trees are keystone structures of forests [13], [21], [22], [74] and their density and distribution can significantly affect the temporal and spatial dynamics of cavity-dependent fauna [20], [26], [62], [75], [76]. In this study, we explored patterns of mortality and collapse among large trees with cavities in forest ecosystems and how key driving factors operate at different spatial scales ranging from the individual tree (e.g. tree species, tree condition, tree height), stand (e.g. stand age, productivity, fire severity) and landscape (fire occurrence, fire severity, climate). We found interactions among some of these drivers such as the tree height and fire severity interaction for tree death (Supplementary Information S2). Notably, some factors such as productivity significantly accelerated tree collapse (Supplementary Information S3) whereas decreasing moisture levels had only marginal effects on increasing tree death. We also have documented the importance of temporal effects with standardized rates of tree death and collapse varying significantly between measurement periods (Figure 4). A particularly significant finding was the ***absence of any recruitment*** of large trees with cavities that might have countered the substantial rates of mortality and collapse among large cavity trees.

Our long-term work has led to several key findings, including: **(1)** Very high rates of mortality among large living trees with cavities on burned sites (Table 2; Table S1; Figure S1); **(2)** High rates of mortality of large living trees with cavities on unburned sites (Table 2; Table S1); **(3)** Losses of a large proportion of large dead trees with cavities on burned sites, even those subject to only moderate severity fire (Table 3; Figure S1); and **(4)** High rates of dead tree collapse on unburned sites (Table 3; Table S1).

Whilst our study focused on an iconic forest ecosystem in south-eastern Australia, as we outline below, this system shares many key features with a range of other forest ecosystems around the world where problems with limited recruitment and subsequent paucity of large trees with cavities have developed or are developing. We therefore argue that new insights from our work will be relevant to forest management of those systems.

### Tree death

High levels of tree death were documented in this investigation both on burned and (surprisingly) unburned sites (Table 2; Table S1). On severely burned sites, almost 80% of the large cavity-bearing trees alive in 1997 were dead 14 years later. The rates of mortality we identified for trees on sites burned at high severity are broadly consistent with those expected for species widely regarded as fire-sensitive, such as Mountain Ash. Notably, our data suggest that many large trees with cavities not killed outright in a major fire event, such as the one which occurred in 2009, will subsequently die in the following 1–2 years (Table 2).

There is strong evidence (P<0.001) for a significant interaction between tree height and fire severity. Taller trees were less likely to die on sites subject to high severity fire. One possible explanation is the relative differences between flame height and tree height. Taller trees extend further above the flame height than short trees and therefore have a reduced risk of being killed during a wildfire [36].

We found that 14% of living, large trees with cavities on unburned sites died between 1997 and 2011. These results are of great concern given that we estimated that the vast majority of large living trees in our study were 150–300 years old and we expected that the majority of them should remain alive for an average of 300–500+ years [55]. Thus, the patterns of mortality we observed have the potential to substantially truncate the lifespan of living trees with cavities (Table 2). Many other studies of long-lived trees suggest that their population dynamics are highly sensitive to temporal changes in mortality rates (e.g. [17], [77], [78]).

The moisture index had only a marginal (P = 0.074) effect on tree death rates. Almost certainly a much longer study, which spanned more measurement periods, would be needed to determine the significance of climate impacts. Elevated tree death rates in response to changes in climatic extremes have been documented elsewhere, including North America and Europe (e.g. [39]–[41], [79]).

### Tree collapse

We recorded high rates of collapse of dead cavity trees on unburned sites along with the high rates of live tree death (Tables 2 and 3). A previous study of tree decay in Mountain Ash forests suggested that rates of tree loss had slowed over the past decade relative to those documented in the 1980s and 1990s [56]. That earlier finding is consistent with the results of the more extensive investigation that we report here. However, the most recent (2010–2011) collapse rates are greater than the low levels observed in 1997–2006 and now resemble those we documented in the 1980s and 1990s (Figure 4). Such patterns of temporal variation contrast with those in other forests where rates of tree fall follow a negative exponential pattern. That is, the rate of collapse slows as an increasing proportion of the tree population is lost (e.g. [49], [80], [81]). Temporal differences in fall rates between studies in the same system might be associated with temporal differences in historical climatic conditions, which can influence tree decay and collapse. For example, drought interspersed with wet periods may contribute to slowing and speeding up of fall rates in unburned areas. Interestingly, we identified a highly significant (P<0.007) relationship between productivity and tree collapse (Supplementary Information S3). For our work, productivity included a measure of moisture and higher collapse rates on more productive sites may be a function of trees being wetter for longer periods and hence being more prone to collapse. They also may be related to larger populations of active decay agents, such as fungi and termites, on more productive sites.

Large trees with cavities were significantly less likely to collapse when present on old growth sites than on sites dominated by 1939 regrowth or 20 year old regrowth (Supplementary Information S3). Two possible reasons may explain this important finding. First, large cavity trees in 1939-aged regrowth forest and 20-year forest are biological legacies remaining after disturbances in previous stands and such older trees may therefore have reached the ending of their standing life. Second, large cavity trees in 1939-aged regrowth forest and 20-year forest may suffer from exposure and greatly altered microclimatic conditions, such as the higher wind speeds and temperatures characteristic of younger regrowth stands [82].

### Fire effects on large trees with cavities

Almost all large, previously dead cavity-bearing trees were lost on burned sites either by direct consumption during the 2009 fire (see Figure S1) or collapsing 1–2 years later (Table 3). This was true even on sites subject to only moderate fire severity (Table 3). We postulate that the decayed wood in large trees with cavities that have been standing dead for a long time may make them particularly vulnerable to fire of any severity. Moreover, even large dead trees with cavities that remain standing after a fire may be highly susceptible to subsequent collapse, which we documented in this study (Table 3).

The more substantial levels of collapse of large cavity trees on moderately burned sites compared to sites subject to a very high severity conflagration was unexpected (Figure 3). This pattern was opposite to that hypothesized at the beginning of this study and we have no ready explanation for this result.

### An ecosystem-wide large tree crisis

**Figure 6:**
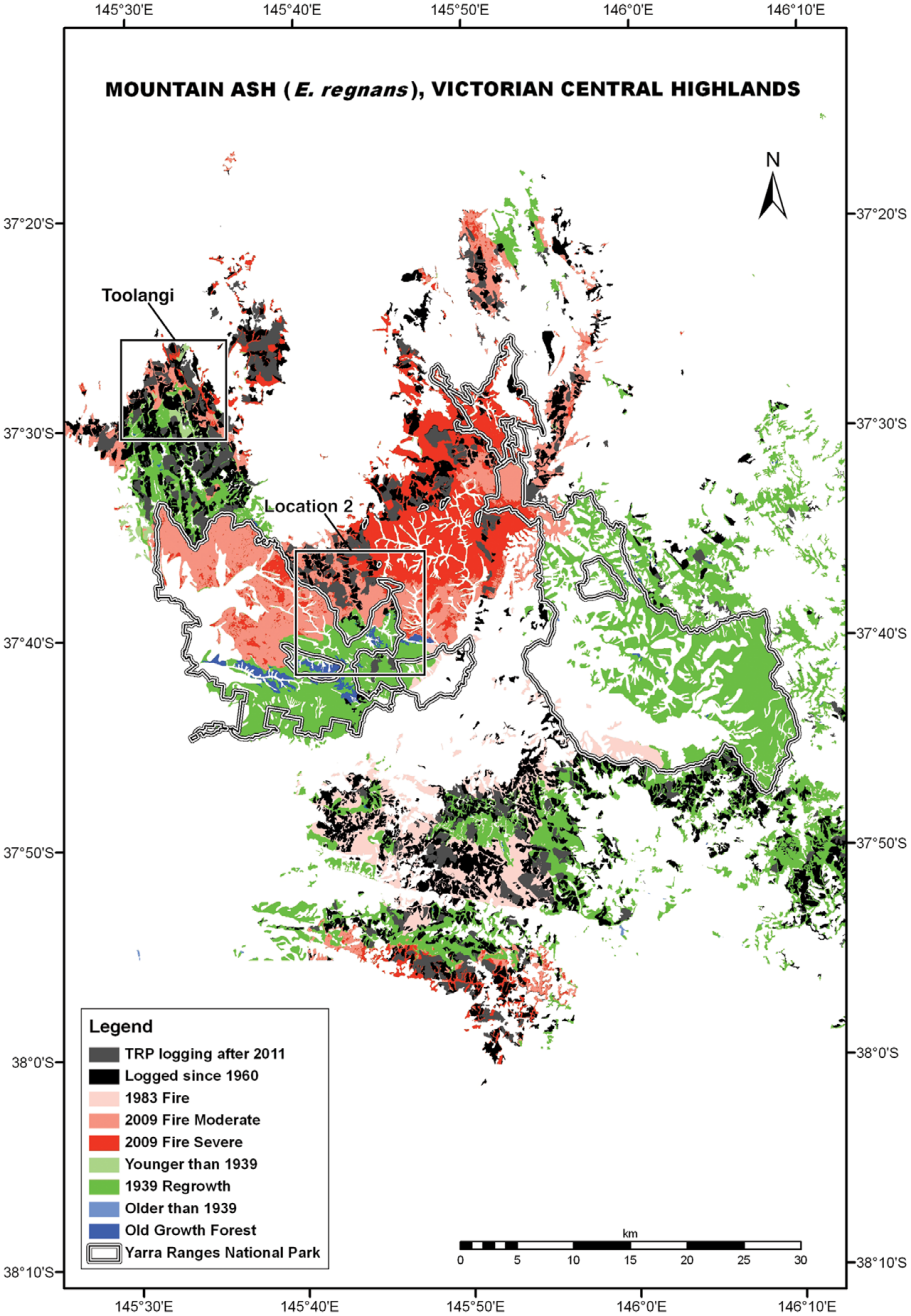
Map of disturbance in Mountain Ash forest in the Central Highlands of Victoria. The map includes the small remaining areas of unlogged and unburned old growth forest – a forest type that encompasses ∼1.16% of the total ash forest resource in the Central Highlands of Victoria. The map shows areas that have been clearcut since the 1960s as well as the 17 655 ha of ash of forest that planned for clearcutting in the coming 5 years under the Victorian Government's Timber Release Plan (TRP) [85]. The two squares are magnified in Figure 7 to show projected abundance of large cavity trees in different disturbance classes.

**Figure 7:**
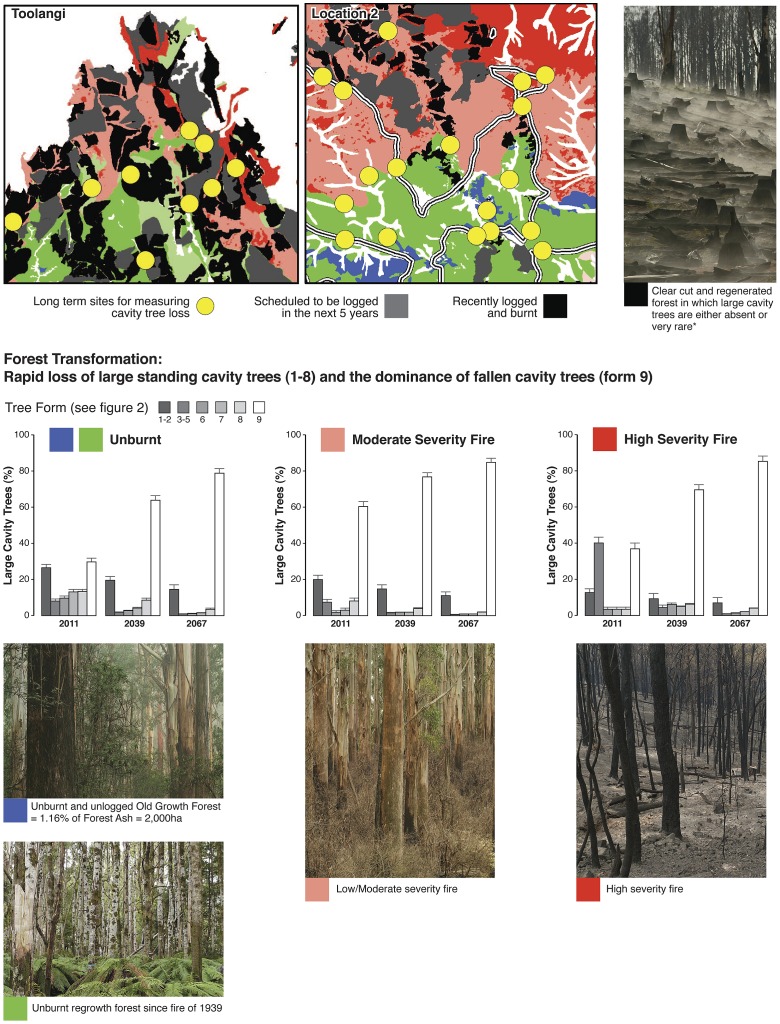
Projected abundance in 2039 and 2067 of large trees with cavities. The projection is for unlogged forest that was either unburned in 2009, subject to moderately severe fire in 2009 or subject to high severity fire in 2009. These projections are based on information contained in Figure 5. We assumed that no large trees with cavities would occur in areas that have been clearcut and slash-burned in the past 40 years or in areas that are planned for clearcutting in the coming five years. We made this assumption because past work [60] has shown that the small numbers of trees retained on harvested sites have a high probability of collapsing after logging. For these projections, we also assumed no further wildfires between 2011 and 2067, and no logging on any of our 156 long-term sites where we are quantifying tree mortality and tree collapse. The star * against the clearcut and regenerated image indicates that we did not study the death and collapse of large trees with cavities on logged sites. This study was not done because large trees with cavities are largely absent or rare in logged sites and/or rapidly collapse in these areas (see text). White areas on the map correspond to non-ash forest.

Our data on tree mortality, rates of tree decay and collapse, and lack of recruitment of new large cavity trees in Mountain Ash forests are strong evidence for rapid development of a regional ecosystem universally depauperate in large cavity-bearing trees. This is illustrated when projections for large trees with cavities in 2039 and 2067 (Figure 5) are overlaid on maps showing the spatial locations of patches of forest subject to different kinds and severities of disturbance (Figures 6 and 7).

**Figure 8:**
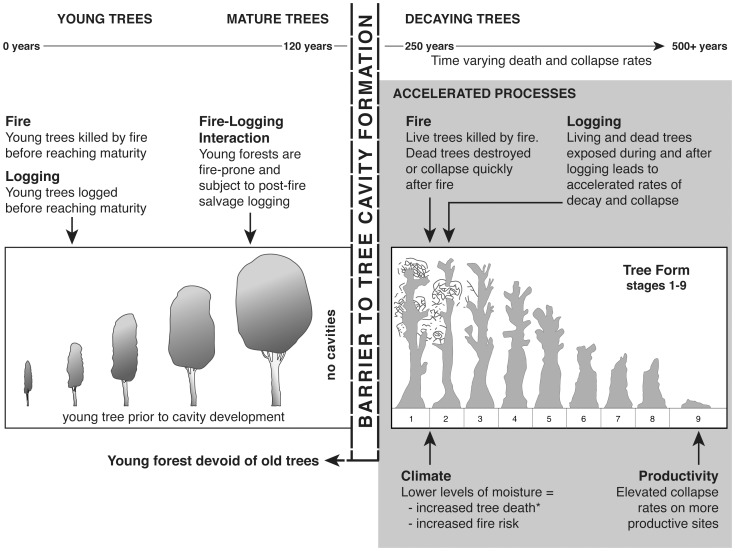
Conceptual model of the key processes influencing large cavity tree death and collapse. The processes include (1) natural disturbance (fire), (2) human disturbance (logging), (3) fire-logging interactions, (4) climate (although the effects of the moisture index on tree death were marginal in this study as indicated by the star * in the model), (5) productivity, and (6) time (as indicated by time varying death and collapse rates). These factors are underpinning accelerated large cavity tree loss and impaired large tree recruitment in Mountain Ash forests.

This crisis in the presence of large cavity-bearing trees is developing in Mountain Ash forests for at least three key, inter-related reasons, which we describe below and illustrate graphically in a new conceptual model (Figure 8). First, large trees are most abundant in unlogged and unburned old-growth stands of Mountain Ash [57]. Unfortunately, less than 1.16% of the 161 200 ha of Mountain Ash forest in the Central Highlands of Victoria is old growth forest. This has resulted from repeated wildfires and extensive clearfell logging, including post-fire salvage logging (Department of Sustainability and Environment, unpublished data). Approximately 99% of Mountain Ash forest is regrowth, and <74 years of age.

Regrowth forests are characterized by a rapidly declining large cavity-bearing tree population because of: **(1)** High rates of mortality among large living cavity trees; **(2)** Extensive losses (57–100%) of large dead cavity-bearing trees that were legacies from stands burned in 1939 or logged in the past few decades and then burned in the 2009 wildfires (Supplementary Information S3). And **(3)** a long interval (50–120 years) before new large cavity-bearing trees will begin to be recruited into existing stands established in 1939 – and longer again in even younger stands (Figure 5).

A second consideration is that wildfires during the next 50–100 years will burn landscapes that are almost completely dominated by young forest. Such fires will **not** produce a pulse of large dead trees with cavities suitable for occupancy by hollow-dependent animals. Such pulses have been characteristic in past fires, like those in 1939, which burned predominately old-growth forest and generated an abundant legacy of large, live fire-scarred trees and large standing dead trees, which subsequently developed cavities [59]. Future fires in the young regrowth forest will generate a legacy of abundant small diameter dead trees that have a short standing period and are incapable of developing significant internal cavities [14]. The only place where large tree recruitment will occur following fires in the coming 50 years will be the tiny area of existing unburned, old-growth Mountain Ash forest that cover only 1.16% of the forest estate.

A third key reason for the impending large cavity tree crisis in Mountain Ash forests is that the widespread young forest devoid of large trees with cavities is susceptible to a feedback process between logging and fire thereby producing an altered fire regime (*sensu* [83]) characterized by increasing fire severity and fire frequency [61]. Thus, because both young burned and regenerating forest and young logged and regenerating forest are fire-prone they are at high risk of re-burning. Fires in the coming 50–100 years would destroy the previously living but recently large class of dead trees that suffered high rates of mortality between 2006 and 2009 on unburned sites – if these dead trees were to remain standing for that long. Forests burned less than 20–30 years after logging or a previous fire may even be subject to a regime shift (*sensu* [84]) and be replaced by a different type of vegetation (e.g. *Acacia* spp. shrub) [33], [35] unless they are artificially seeded. In summary, additional fires in the future will kill existing living large trees, consume existing large dead trees, and will considerably set back the time until recruitment of new cohorts of large trees (Figure 8).

We suggest that the large cavity tree crisis in Mountain Ash forests could be prolonged – possibly exceeding 100–150 years in large parts of the Central Highlands region. This is because within the existing 40 000 ha of unburned and unlogged 1939 regrowth Mountain Ash forest, it will take at least another 50–120 years before existing ∼73 year-old trees become large and old enough for cavities suitable for use by cavity-dependent animals like arboreal marsupials to develop. For the deficit in large cavity trees to be rectified after that 50–120 year period there must be no fire, no traditional (clearcut) logging, and no salvage logging. This is an unrealistic prospect given there has been five major and three substantial fires in the past ∼100 years making the complete absence of fire for the next 50–120 years unlikely. In addition, over the coming five years the Government of Victoria has committed itself to log an additional 17 665 ha covering 412 new cutblocks (averaging ∼45 ha each) [85] thereby putting considerable harvesting pressure on existing areas of unlogged and unburned 1939 regrowth forest (Figures 6 and 7).

### Ecological consequences of a large tree crisis

Significant negative ecological consequences will arise from the Mountain Ash-wide absence of large cavity trees. These consequences include: **(1)** Simplified stand structures (*sensu* [86]), which will lack suitable habitat for many native biota [37], [87], [88]. **(2)** Reduced levels of carbon storage [8]. And **(3)** impaired key ecosystem processes like the recruitment of large logs to the forest floor [7], [89]. In the particular case of Mountain Ash forests, a paucity of large-diameter dead trees will deplete the nesting and denning resources required by ∼40 species of cavity-dependent vertebrates in these ecosystems.

Past work has highlighted strong relationships between the abundance of large trees with cavities and the presence and abundance of many species including the endangered Leadbeater's Possum [33], [90]. This species, which typically nests and dens in large dead trees [91] (forms 6–8 in Figure 2), may be especially disadvantaged by the rapid rates of collapse by large dead trees with cavities (Table 3). In addition, key patterns of behaviour like denswapping between multiple large cavity trees exhibited by almost all members of cavity-dependent animal communities like arboreal marsupials (reviewed by [14]) also will be substantially curtailed in highly simplified stands where large trees with cavities are rare.

### Characteristics of forests that make them prone to a shortage of large trees with cavities

Large cavity trees exhibit strong temporal patterns in occurrence, abundance, and condition [20], [49], [50] but the recent worldwide decline of large old trees has become a topic of conservation concern in an array of different ecosystems worldwide [1], [11], [16]–[19], [30], [92], [93]. We suggest that Mountain Ash forests have a suite of characteristics that make them particularly vulnerable to a decline in abundance of large trees and these characteristics are shared with many forest ecosystems around the world such as the Douglas Fir (*Pseudostuga menziesii*) forests of western North America [3], [94], boreal forests of North America and Europe [2], [37], [95], and some kinds of tropical forests [25], [92], [96]. These characteristics include: **(1)** The death and/or removal of trees *en masse* as a result of a natural disturbance event (e.g. stand-replacing wildfire or windstorms) [86], [92]. **(2)** A prolonged period of extensive and/or intensive human disturbance such as logging [25], [30], [37], [97]. **(3)** A prolonged period (typically >100 years) for trees to mature and attain a large size. **(4)** An asymmetry between the rapidity with which large trees can be removed over extensive areas and the time that must elapse for them to regrow and provide key structural features like cavities [20], [30], [37], [74], [98]. And **(5)** Predicted changes in climatic conditions such as increasing temperatures and reduced rainfall. These, in turn, alter natural disturbance regimes such as wildfires [99], [100], windstorms and hurricanes [101] or an increased prevalence of pests and diseases [102], [103] – all of which can trigger mass tree mortality events [39], [41], [104], [105]. Hence, the insights we present about the dynamics of large trees are relevant to tackling problems with the paucity of large trees in many other ecosystems in particular, as we discuss below, a shift in policy and management actions.

### Management and policy options

We suggest that immediate, dramatic changes in forest policy and associated management actions are essential to tackle the large tree crisis developing within Mountain Ash forests. The major drivers of the problem have been extensive past logging, particularly traditional intensive clearcut harvesting undertaken over the past four decades, recurrent high-severity wildfires, and post-fire salvage logging. These drivers are not independent as, for example, traditional green-tree logging can make moist forests more fire-prone [61], salvage logging (by definition) follows disturbances like wildfire [106], and salvage logging can increase fire-proneness of forests [107].

New policies and management actions should better protect the recruitment process for large trees with cavities. These include: **(1)** The continued protection of all remaining previously unlogged and unburned (old growth) forest. **(2)** The continued exclusion of salvage logging in old growth forest that was burned in the 2009 wildfires because the large dead trees created by burning of old growth stands are critical biological legacies for biodiversity and carbon storage in subsequent regenerating stands. **(3)** The protection of substantial parts of the ∼40 000 ha of remaining unburned areas of 1939 regrowth forest because these ∼73-year old stands are now the next nearest existing age class to old growth forest. **(4)** If recommendation #3 takes some years (which is not desirable), then any continued logging operations must be excluded from those areas where there are existing large trees scattered throughout forests of 1939 regrowth. This is because of the very high habitat value of any remaining large trees that remain standing, and the greatly accelerated rate of tree mortality and collapse that occurs among retained trees when the stands surrounding them are cut down [60], [77]. In addition, logging operations should be excluded from areas that have previously been identified (see [36]) as having a high probability of being fire refugia. We also recommend that any activities that might make the forest more fire-prone should be curtailed. For example, roads are well known to be key point sources of fire [108] and the construction of new roads in currently roadless areas should be avoided.

The policy recommendations we have outlined above will require a comprehensive restructuring of the timber industry in the Central Highlands of Victoria. At a State Government level, this will require radically reducing sustained yields and developing exit strategies and financial support packages for people currently directly employed in the timber industry. At a national level, this will require an overhaul of the Regional Forest Agreement between the Australian Government and the State Government [109].

Finally, a paucity of large trees corresponds to a paucity of cavities, suggesting that strategies are needed to supplement and/or accelerate hollow development. One strategy is to install artificial cavities such as nest boxes [26], [110], [111] and this has sometimes been remarkably successful. For example, nest boxes added to forests in Germany throughout the 1950s resulted in a 5–20 fold population increase in some bird species [112]. Artificial cavities have resulted in other spectacular population recoveries of birds such as three species of Bluebirds (*Sialia* spp.), and the Wood Duck (*Aix sponsa*) in North America [113]. In addition, nest boxes have been added to logged forests (where trees with hollows had been removed) with significant recoveries of populations of some cavity-dependent species (e.g. [114], [115]). However, past work has highlighted the ineffectiveness of nest boxes in Mountain Ash forests [116]. An alternative to nest boxes in Mountain Ash forests might be to deliberately injure trees to promote cavity formation using techniques like tree girdling that have met with some success in Northern Hemisphere forests [3], [117], [118]. These approaches have remained untried in Australian hardwood forest ecosystems but urgently need to be trialed given the major crisis developing with large trees with cavities in Mountain Ash forests.

### Caveats

Our study focused on the decay, mortality and collapse of large cavity trees – trees which we have defined in a precise manner and then carefully and repeatedly re-measured over a prolonged period using a standardized field sampling protocol. Although we did not find evidence of the recruitment of new large cavity trees on any of our 156 field sites between 1998 and 2011, as outlined above, we are aware that some new trees may have been missed because of the ground-based protocol that we have employed. Calibration studies are needed to determine relationships between ground-based measurement of the numbers of cavities in Mountain Ash and the actual numbers of suitable cavities in such trees (e.g. [25], [62]). Methods like dissections of trees [105] and climbing trees to confirm the presence of cavities would be needed to develop appropriate calibration measures.

A second important caveat associated with our work was that projections of the future abundance of large cavity trees are likely to be highly optimistic. This was because we made a number of simplifying assumptions for the projections including a paucity of future fire and logging over the coming 50–120 years and that the age profile of our 156 field sites was representative of Mountain Ash forests across the Central Highlands of Victoria. These assumptions mean that the on-ground paucity of large cavity trees in Mountain Ash forests is likely to be more severe than indicated in our projections in Figures 5 and 7. Hence, the fate of cavity-dependent species like Leadbeater's Possum is likely to be more perilous than suggested by the current projections of the future availability of large cavity trees.

## Supporting Information

Figure S1 **Collapsed tree on an unburned site, and fire-consumed tree on a site burned at moderate severity.**
(DOCX)

Supplementary Information S1 **Statistical methods – further details.**
(DOC)

Supplementary Information S2 **Generalized Linear Mixed Model for tree death.**
(DOC)

Supplementary Information S3 **Generalized Linear Mixed Model for tree collapse.**
(DOC)

Table S1 **Transition probability matrices for fire severity classes computed for 1997–2011 and 1993–2007.**
(DOCX)
