# Supplementary material for: Interacting Factors Driving a Major Loss of Large Trees with Cavities in a Forest Ecosystem
Source: PLoS One. 2012 Oct 5;7(10):e41864. doi: 10.1371/journal.pone.0041864 (PMC3465306; doi:10.1371/journal.pone.0041864)

**Figure S1**

**(A) Collapsed tree on an unburned site (Photo: David Lindenmayer)**


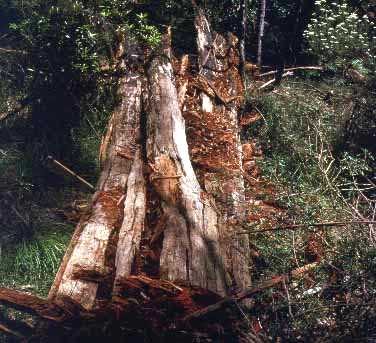


**(B) Fire-consumed tree on a site burned at moderate severity (Photo: David Blair)**


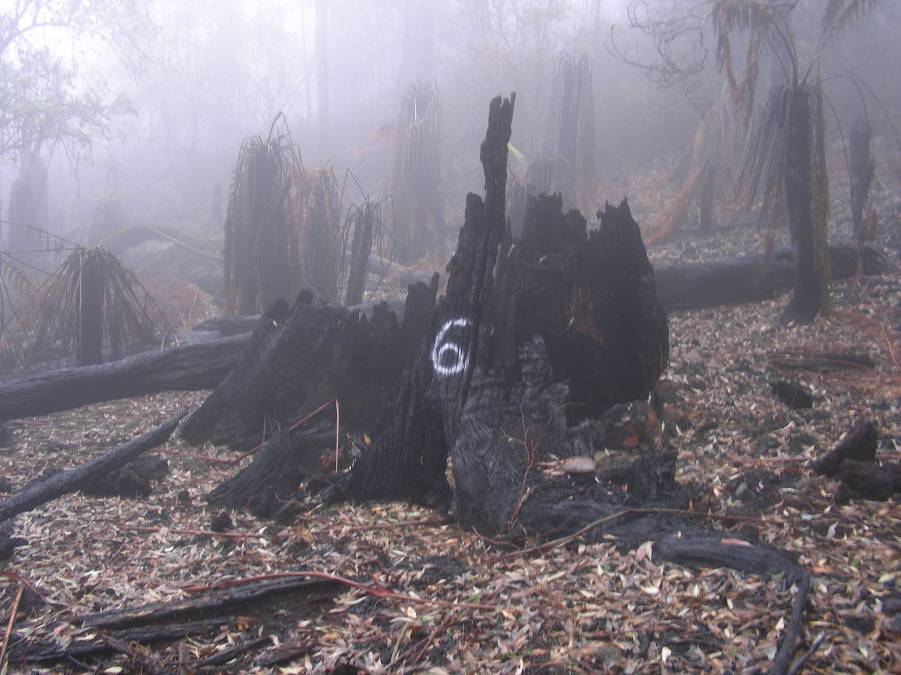

Supplement: Figure S1 — Collapsed tree on an unburned site, and fire-consumed tree on a site burned at moderate severity. (DOCX) [file pone.0041864.s001.docx]
