# Supplementary material for: Interacting Factors Driving a Major Loss of Large Trees with Cavities in a Forest Ecosystem
Source: PLoS One. 2012 Oct 5;7(10):e41864. doi: 10.1371/journal.pone.0041864 (PMC3465306; doi:10.1371/journal.pone.0041864)
Supplement: Supplementary Information S1 — Statistical methods – further details. (DOC) [file pone.0041864.s002.doc]

**Supplementary Information S1: Statistical methods – further details**

The survival function is denoted by $S(t)$ and related quantity known as the hazard function as defined by:

.

That is, the hazard function is the probability of death in the interval $[t,t+\Delta t)$ given that one has survived to time t. The function $h(t)$ is also known as the hazard rate, instantaneous death rate, or the intensity rate. It can be shown that the hazard function and survival function can be related via the following equation (see [1]):

$$h(t)=-\frac{d}{dt}\left\{\log S(t)\right\}$$

which then allows relation of the integrated hazard directed to the survival function as follows:

$H(t)=\int_{0}^{t} h(u)du=-\log\left\{S(t)\right\}$.

This equation enables us to write the model in terms of the integrated hazard as follows:

$$\log\left(-\log\left(1-p_{i}\right)\right)=l_{i}+\log\left\{H(t)\right\},$$

where $l_{i}$is the linear predictor. Thus, we can interpret the model parameters as integrated hazard ratios.

To adjust for the differing lengths of observation period we employed the following strategy to adjust the integrated hazard ratios, a similar approach was described in [2]. The integrated hazard ratio was adjusted by the ratio of the corresponding lengths of observation. Let $t_{1}$and $t_{2}$ be length of observation for the two time periods of interest. Therefore the integrated hazard ratios are $H\left(t_{1}\right)$ and$H\left(t_{2}\right)$. If the hazards in the two time intervals were proportional to the length of the interval we would expect the following ratio to be one:

$$\frac{H\left(t_{2}\right)\times t_{1}}{H\left(t_{1}\right)\times t_{2}}$$

We report these adjusted integrated hazard ratios in tables provided in Supplementary Information S2 and S3.

**References**

1. Collett D (2003) Modelling survival data in medical research. Chapman & Hall/CRC.

2. Lindenmayer DB, Wood JT (2010) Long-term patterns in the decay, collapse, and abundance of trees with hollows in the mountain ash (Eucalyptus regnans) forests of Victoria, southeastern Australia. Can J Forest Res 40: 48–54.
