# Supplementary material for: Interacting Factors Driving a Major Loss of Large Trees with Cavities in a Forest Ecosystem
Source: PLoS One. 2012 Oct 5;7(10):e41864. doi: 10.1371/journal.pone.0041864 (PMC3465306; doi:10.1371/journal.pone.0041864)
Supplement: Supplementary Information S2 — Generalized Linear Mixed Model for tree death. (DOC) [file pone.0041864.s003.doc]

**Supplementary Information S2: Generalized Linear Mixed Model for tree death (see Methods for details).**

**Table 1.**

Note the estimates and significance levels for period are not adjusted for the period length (see Tables 2 and 3 below). Variables not shown include aspect, slope, age, elevation and diameter which had p-values ranging from 0.14 to 0.47. In addition, other variables such as TWI were eliminated in the early stages of our analyses because of high levels of multi-colinearity.

| Constant | -5.408 | 0.733 |  |
| --- | --- | --- | --- |
| period 1997-2006 | 0 | -- | **<0.001** |
| period 2006-2009 | 2.791 | 0.573 |  |
| period 2009-2010 | 0.688 | 0.947 |  |
| period 2010-2011 | 0.005 | 1.231 |  |
| Moderate Fire 2006-2009 | 1.131 | 0.504 | 0.025 |
| Severe Fire 2006-2009 | 2.339 | 0.504 | * |
| Moderate Fire 2009-2010 | 1.914 | 1.075 | 0.075 |
| Severe Fire 2009-2010 | 4.590 | 0.926 | **<0.001** |
| Moderate Fire 2010-2011 | 2.321 | 1.390 | 0.095 |
| Severe Fire 2010-2011 | 5.345 | 1.227 | **<0.001** |
| Height | -0.078 | 0.012 | * |
| Alpine Ash | 0 | -- | **<0.001** |
| Mountain_Ash | 0.217 | 0.610 |  |
| Mountain_Gum | -0.218 | 0.675 |  |
| Shining_Gum | -2.327 | 0.762 |  |
| Unknown Species | 0.310 | 0.998 |  |
| Severe Fire 2006-2009 x Height | 0.044 | 0.018 | 0.017 |

**Table 2.**

| **Integrated Hazard/ Ratio of Interval** | **1997-2006** | **2006-2009** | **2009-2010** |
| --- | --- | --- | --- |
| 2006-2009 | 48.87 (P<0.001) |  |  |
| 2009-2010 | 17.91 (P=0.002) | 0.37 (P<0.001) |  |
| 2010-2011 | 9.04 (P=0.074) | 0.19 (P<0.001) | 0.50 (P=0.615) |

**Table 3.**

| **Integrated Hazard** | **Moderate/No Fire** | **Severe Fire/No Fire** | **Severe/Moderate** |
| --- | --- | --- | --- |
| 2006-2009 | 3.10 (P=0.025) | 10.37 (P<0.001) | 3.35 (P=0.025) |
| 2009-2010 | 6.78 (P=0.075) | 98.45 (P<0.001) | 14.52 (P=0.001) |
| 2010-2011 | 10.18 (P=0.095) | 209.61 (P<0.001) | 20.58 (P=.001) |
