# Supplementary material for: Interacting Factors Driving a Major Loss of Large Trees with Cavities in a Forest Ecosystem
Source: PLoS One. 2012 Oct 5;7(10):e41864. doi: 10.1371/journal.pone.0041864 (PMC3465306; doi:10.1371/journal.pone.0041864)
Supplement: Supplementary Information S3 — Generalized Linear Mixed Model for tree collapse. (DOC) [file pone.0041864.s004.doc]

**Supplementary Information S3: Generalized Linear Mixed Model for tree collapse (see Methods for details).**

**Table 1.**

Note the estimates and significance levels for period are not adjusted for the period length (see Tables 2 and 3 below). Variables not shown include aspect, slope, and diameter which had p-values ranging from 0.57 to 0.79. In addition, other variables such as TWI were eliminated in the early stages of our analyses because of high levels of multi-colinearity.

| **Variable** | **Estimate** | **SE** | **P** |
| --- | --- | --- | --- |
| Constant | -5.429 | 0.360 |  |
| period 1997-2006 | 0 | -- | **<0.001** |
| period 2006-2009 | 1.301 | 0.164 |  |
| period 2009-2010 | -0.490 | 0.284 |  |
| period 2010-2011 | -0.443 | 0.284 |  |
| Moderate Fire 2006-2009 | 1.833 | 0.163 | **<0.001** |
| Severe Fire 2006-2009 | 1.878 | 0.214 | **<0.001** |
| Moderate Fire 2009-2010 | -0.994 | 1.063 | 0.349 |
| Severe Fire 2009-2010 | 0.674 | 0.594 | 0.257 |
| Moderate/Severe Fire 2009-2010 | -0.933 | 0.770 | 0.226 |
| Old Growth | 0 | -- | **0.009** |
| 1939 regrowth | 0.671 | 0.220 |  |
| Young Forest | 0.574 | 0.286 |  |
| Alpine Ash | 0 | -- | **0.002** |
| Mountain Ash | 0.203 | 0.237 |  |
| Mountain Gum | 0.436 | 0.392 |  |
| Shining Gum | 0.051 | 0.488 |  |
| Unknown Species | 0.735 | 0.244 |  |
| Form 1-2 | 0 | -- | **<0.001** |
| Form 3-5 | 1.871 | 0.209 |  |
| Form 6 | 2.170 | 0.211 |  |
| Form 7 | 2.153 | 0.208 |  |
| Form 8 | 2.747 | 0.237 |  |
| Avg Jan-Mar Moisture Index | -0.137 | 0.771 | 0.074 |

**Table 2.**

| **Integrated Hazard/**  **Ratio of Interval** | **1997-2006** | **2006-2009** | **2009-2010** |
| --- | --- | --- | --- |
| 2006-2009 | 11.02 (P<0.001) |  |  |
| 2009-2010 | 5.51 (P<0.001) | 0.50 (P<0.001) |  |
| 2010-2011 | 5.78 (P<0.001) | 0.52 (P<0.001) | 1.05 (P=0.891) |

**Table 3.**

| **Integrated Hazard** | **Moderate/No Fire** | **Severe Fire/No Fire** | **Severe/Moderate** |
| --- | --- | --- | --- |
| 2006-2009 | 6.25 (P<0.001) | 6.54 (P<0.001) | 1.05 (P=0.834) |
| 2009-2010 | 0.37 (P=0.349) | 1.96 (P=0.257) | 5.30 (P=0.151) |
| 2010-2011 | 0.39 (P=0.226) |  |  |
