# Supplementary material for: Interacting Factors Driving a Major Loss of Large Trees with Cavities in a Forest Ecosystem
Source: PLoS One. 2012 Oct 5;7(10):e41864. doi: 10.1371/journal.pone.0041864 (PMC3465306; doi:10.1371/journal.pone.0041864)
Supplement: Table S1 — Transition probability matrices for fire severity classes computed for 1997–2011 and 1993–2007. (DOCX) [file pone.0041864.s005.docx]

**Table S1. Transition probability matrices for fire severity classes computed for 1997-2011 and 1993-2007**

|  |  | **2011 Tree Form** | | | | | |  |
| --- | --- | --- | --- | --- | --- | --- | --- | --- |
| **Fire Severity** | 1997 tree form | 1-2 | 3-5 | 6 | 7 | 8 | 9 | No. of Trees |
| None | 1-2 | 86.0 | 3.2 | 2.7 | 1.6 | 0.5 | 5.9 | 186 |
|  | 3-5 |  | 31.1 | 24.6 | 4.3 | 8.0 | 31.9 | 138 |
|  | 6 |  |  | 22.6 | 27.4 | 9.4 | 40.6 | 106 |
|  | 7 |  |  |  | 30.5 | 27.2 | 42.4 | 151 |
|  | 8 |  |  |  |  | 47.6 | 52.4 | 42 |
|  | No. of trees | 160 | 49 | 63 | 84 | 83 | 184 | 623 |
| Moderate | 1-2 | 63.2 | 13.8 | 2.3 | 1.1 | 2.3 | 17.2 | 87 |
|  | 3-5 |  | 19.0 | 0.0 | 7.1 | 11.9 | 61.9 | 42 |
|  | 6 |  |  | 4.3 | 0.0 | 8.7 | 87.0 | 69 |
|  | 7 |  |  |  | 5.6 | 12.7 | 81.7 | 71 |
|  | 8 |  |  |  |  |  | 100.0 | 7 |
|  | No. of trees | 55 | 20 | 5 | 8 | 22 | 166 | 276 |
| Severe | 1-2 | 20.6 | 61.7 | 2.8 | 0.7 | 2.1 | 12.1 | 141 |
|  | 3-5 |  | 13.9 | 8.3 | 11.1 | 5.6 | 61.1 | 36 |
|  | 6 |  |  | 3.6 | 0.0 | 7.1 | 89.3 | 28 |
|  | 7 |  |  |  | 15.0 | 5.0 | 80.0 | 20 |
|  | 8 |  |  |  |  |  | 100.0 | 5 |
|  | No. of trees | 29 | 92 | 8 | 8 | 8 | 85 | 230 |
|  |  | **2007 Tree Form** | | | | | |  |
|  | 1993 tree form | 1-2 | 3-5 | 6 | 7 | 8 | 9 | No. of Trees |
| Background | 1-2 | 81.2 | 5.8 | 2.9 | 0.0 | 2.9 | 7.2 | 69 |
|  | 3-5 |  | 52.9 | 23.5 | 5.9 | 0.0 | 17.6 | 17 |
|  | 6 |  |  | 26.9 | 19.2 | 15.4 | 38.5 | 26 |
|  | 7 |  |  |  | 16.7 | 44.4 | 38.9 | 18 |
|  | 8 |  |  |  |  | 38.6 | 61.4 | 44 |
|  | No. of trees | 56 | 13 | 13 | 9 | 31 | 52 | 174 |
